# Body mass index increases the lymph node metastasis risk of breast cancer: a dose-response meta-analysis with 52904 subjects from 20 cohort studies

**DOI:** 10.1186/s12885-020-07064-0

**Published:** 2020-06-29

**Authors:** Junyi Wang, Yaning Cai, Fangfang Yu, Zhiguang Ping, Li Liu

**Affiliations:** 1 College of Public Health, Zhengzhou University, No.100 Science Avenue, Zhengzhou City, 450001 Henan Province China; 2 School of Basic Medical Sciences, Zhengzhou University, Zhengzhou, Henan China

**Keywords:** Body mass index, Metastasis, Breast cancer, Dose-response relationship, Meta-analysis

## Abstract

**Background:**

Since body mass index (BMI) is a convincing risk factor for breast cancer, it is speculated to be associated with lymph node metastasis. However, epidemiological studies are inconclusive. Therefore, this study was conducted to investigate the effect of BMI on the lymph node metastasis risk of breast cancer.

**Methods:**

Cohort studies that evaluating BMI and lymph node metastasis in breast cancer were selected through various databases including PubMed, PubMed Central (PMC), Web of science, the China National Knowledge Infrastructure (CNKI), Chinese Scientific Journals (VIP) and Wanfang Data Knowledge Service Platform (WanFang) until November 30, 2019. The two-stage, random effect meta-analysis was performed to assess the dose-response relationship between BMI and lymph node metastasis risk. Between-study heterogeneity was assessed using *I*^*2*^. Subgroup analysis was done to find possible sources of heterogeneity.

**Results:**

We included a total of 20 studies enrolling 52,904 participants. The summary relative risk (*RR*) (1.10, 95%*CI*: 1.06–1.15) suggested a significant effect of BMI on the lymph node metastasis risk of breast cancer. The dose-response meta-analysis (*RR =* 1.01, 95%*CI*: 1.00–1.01) indicated a positive linear association between BMI and lymph node metastasis risk. For every 1 kg/m^2^ increment of BMI, the risk of lymph node metastasis increased by 0.89%. In subgroup analyses, positive linear dose-response relationships between BMI and lymph node metastasis risk were observed among Asian, European, American, premenopausal, postmenopausal, study period less than 5 years, and more than 5 years groups. For every 1 kg/m^2^ increment of BMI, the risk of lymph node metastasis increased by 0.99, 0.85, 0.61, 1.44, 1.45, 2.22, and 0.61%, respectively.

**Conclusion:**

BMI significantly increases the lymph node metastasis risk of breast cancer as linear dose-response reaction. Further studies are needed to identify this association.

## Background

Breast cancer is one of the most common malignant tumors among females worldwide. According to the International Agency for Research on Cancer’s GLOBOCAN 2018 [1], breast cancer was the second most common cancer only after lung cancer and the most frequent cancer among women with an estimated 2.09 million new cases diagnosed worldwide, making up 11.6% of all new cancer cases. Relative to cases, breast cancer ranked as the fourth cause of death from cancer overall (627 thousands), accounting for 6.6% of all cancer deaths. In China, it was estimated that there were 67,328 new breast cancer cases (16.3% of all cancer cases) and 16,178 deaths (7.8% of all deaths) occurred in 2015 [2]. In addition, over the past decades, the prevalence of breast cancer is rising and getting younger gradually [3–5], which has caused serious economic burden and become an important global public health issue.

Although the rise in obesity and overweight showed some signs of leveling off, data from several countries indicated that obesity has become a worldwide epidemic [6]. Based on linear time trend analysis, a 33% increase in obesity (body mass index, BMI ≥ 30 kg/m^2^) prevalence was estimated, and obesity rates will be exceed 50% by 2030 [7]. It was regarded as a modifiable lifestyle risk factor for several chronic diseases in a growing body of literature, such as coronary heart disease [8], hypertension [9], type 2 diabetes mellitus [10], hyperlipidemia [11], stroke [12] and some cancers [13, 14]. Among them, several studies have found that overweight or obese women have an increased risk of breast cancer as compared to normal weight women, especially in postmenopausal women. A case-control study [15] conducted in Iran reported that obese postmenopausal women had a threefold increased risk of breast cancer (odds ratio, *OR* = 3.21, 95% *CI*: 1.15–8.47). In a pooled analysis [16] of eight representative large-scale cohort studies, the increased risk of breast cancer with higher BMIs was confirmed among Japanese postmenopausal women. Yanzi Chen’s [17] dose-response meta-analysis was performed on BMI and breast cancer incidence, which showed that the breast cancer risk increased by 3.4% for every 1 kg/m^2^ increment of BMI in postmenopausal women. Furthermore, women who are obese with breast cancer diagnosis were reported to have greater disease mortality, higher recurrence rate and adverse overall and disease-free survival [18, 19]. So obesity also plays an important role in the prognosis of breast cancer.

Despite accumulated evidence that obesity may increase breast cancer risk, question remain, whether obesity is associated with lymph node metastasis, the most common form of metastasis in breast cancer? However, there was limited study focused on the relationship between obesity and lymph node metastasis in breast cancer, and the conclusions were inconsistent. For example, in a retrospective review of 1352 breast cancer patients [20], obese patients were more likely to have lymph node metastases compared with non-obese patients (*P* = 0.026). In another study [21] supporting this viewpoint, obesity was associated with increased number of involved axillary nodes (*P* = 0.003). On the contrary, Yadong Cui’s [22] case series study found that there was no statistically significant association between BMI and axillary node involvement (adjusted *OR* = 1.28, 95% *CI*: 0.90–1.81). Therefore, the present dose-response meta-analysis was conducted to investigate the association between obesity, as measured by BMI, and lymph node metastasis in breast cancer, and sub-analyses by different areas, menopausal status, study period were done to explore potential factors that influence the associations deeply.

## Methods

### Search strategy

In this study, we searched PubMed, PubMed Central (PMC), Web of science and Chinese academic databases including the China National Knowledge Infrastructure (CNKI), VIP database of Chinese Scientific Journals (VIP) and Wanfang Data Knowledge Service Platform (WanFang) for publications on the association between BMI and lymph node metastasis in breast cancer in humans up to November 30, 2019. The following combination of keywords was used to identify studies from electronic databases: (obesity OR “body mass index” OR BMI) AND (“breast cancer”) AND (“metastasis”). To avoid missing any relevant studies, all reference lists of eligible articles and related reviews were searched for additional publications. We did not include unpublished documents and grey literature, such as conference abstracts, theses (including dissertations) and patents.

### Study selection

Studies were included according to the following criteria: (1) full-text articles were available as Chinese or English language; (2) study design was a cohort study; (3) the height and weight of patients were measured at the time of diagnosis; (4) studies had BMI categories of no fewer than three, and provided the number of cases for each BMI category; (5) studies reported the metastasis type of patients, such as lymph node metastasis, positive lymph nodes and so on. If more than one publication of a given study exists, only the publication with higher number participants was included.

### Data extraction

All potential relevant publications were inserted in EndNote X8 software. Then, qualified studies were obtained for full-text screening. After the final evaluation, the authors extracted and recorded the required data: name of the first author; year of publication; country of origin; age (range) of study population; study period; intervals of each BMI category; cases number of each category and so on.

### Quality assessment

Using the Newcastle-Ottawa’s Scale (NOS), the quality of the included studies were assessed. This scale ranges from 0 to 9 stars and awards four stars for selection of study participants, two stars for comparability of studies, and three stars for the adequate ascertainment of outcomes, and each item is assigned with a star if a study meets the criteria. We considered a study to be of high quality if its NOS score was more than six stars.

Study selection, data extraction, and quality assessment were done by two independent reviewers, and any controversies across selecting eligible articles were resolved by mutual discussion.

### Statistical analysis

The relative risk (*RR*) and its 95%*CI* were considered as the effect size of all studies. For the highest versus lowest category meta-analysis, the risk estimates for the highest compared with the lowest categories of BMI was combined using the DerSimonian and Laird random-effects model [23]. For the dose-response meta-analysis, the dosage value corresponding to each BMI was the median or mean of the upper and lower boundaries. When the lowest or the highest category was open-ended, we assumed that the open-ended interval length was same as the adjacent interval [24, 25].

For non-linear dose-response relation, the covariance-adjusted multiple variables regression model was used to estimate and test the overall effect of curvilinear dose-responses. For linear dose-response relationship, a slope for each study was estimated as the first step, then derived an overall estimates by weighted average of the individual slopes [26].

Heterogeneity among studies was assessed by I-square (*I*^*2*^) statistic. An *I*^*2*^ above 50% indicated high heterogeneity, and a random effect model was implemented. Predefined subgroup analyses based on area, menopausal status, study period and study population were conducted to detect potential sources of heterogeneity. To explore the influence of each study on the pooled effect size, a sensitivity analysis was used by omitting one study at a time. Publication bias was identified with the Begg’s rank correlation test and Egger’s regression test [27, 28]. All statistical analyses were performed using Stata software version 14.0 (Stata Corp, College Station, TX, USA). Statistical significance level was set at α = 0.05, except publication bias or heterogeneity test with α = 0.10.

## Results

### Literature screening results

**Fig. 1:**
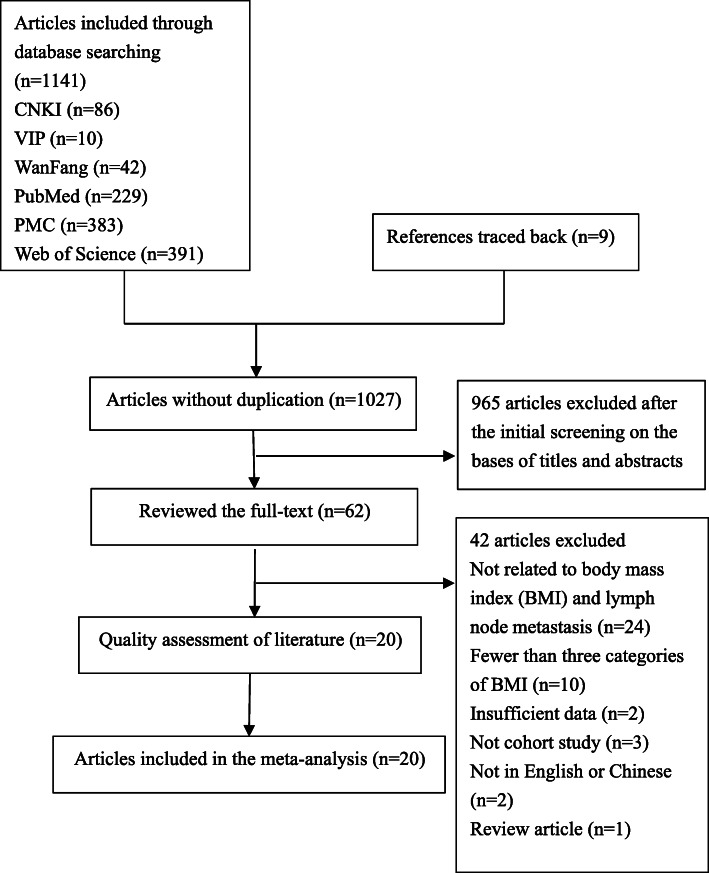
Flow chart of literature retrieval and selection for this meta-analysis (CNKI: China National Knowledge infrastructure; VIP: VIP database of Chinese Scientific Journal; WanFang: Wanfang Data Knowledge Service Platform; PMC: PubMed Central)

From the preliminary literature search, a total of 1141 articles were identified, with 9 references traced back. After excluding 123 de-duplicated publications, we read 1027 titles and abstracts. Upon the exclusion of 965 clearly irrelevant records, we obtained 62 full-text articles for further assessment. Finally, a total of 20 articles were initially included in this meta-analysis. Among them, there were one Chinese article and 19 English articles. A detailed description of how studies were selected is presented in Fig. 1.

### Characteristics and quality assessment

There were total 20 [29–48] articles included, all of which were cohort studies with a sample size of 52,904 people. Among the 20 studies, three studies were conducted in Asia, eight in Europe, eight in America and one from the International Breast Cancer Study Group, which covering the population from the whole world. Besides, four studies provided information on premenopausal and postmenopausal women separately, one study provided data on premenopausal women, and two studies provided data on postmenopausal women only. In terms of study period, there were six studies less than or equal to 5 years, and 14 studies more than 5 years. As for study population, two studies focused on triple-negative breast cancer (TNBC) patients. NOS scale was used to evaluate the included articles with score ranged from 6 to 8. The characteristics and quality score of the individual studies are shown in Table 1.

**Table 1:** The characteristics of studies included in this meta-analysis

Author	Year	Country	Age (range)	Study period	The categories of BMI	The number of metastatic tumors	The number of non-metastatic tumors	NOS
Xiaoyao Zhang	2014	China	53 (27-92)	2010.1-2012.11	BMI <18.5 (underweight)/ 18.5-22.9 (normal)/ 23-24.9 (overweight)/ 25-29.9 (obese)/ BMI≥30 (severe obese)	2/27/21/85/25	7/56/51/115/35	6
Nicoletta Biglia	2013	Italy	45/65	1999.1-2009.12	BMI < 19 (underweight)/ 19-24.9 (normal)/ 25-29.9 (overweight)/ BMI≥30 (obese)	20/141/49/29 (premenopausal) 20/247/217/97 (postmenopausal)	37/200/44/20 (premenopausal) 35/372/243/125 (postmenopausal)	7
Orsolya Hankó-Bauer	2017	Romania	58.29 (27-80) 52.81/60.38/62.8	2012-2015	BMI < 25 (normal weight)/ 25-29.9 (overweight)/ BMI≥30 (obese)	32/40/40	54/48/31	6
Ahmad Kaviani	2013	Iran	49.62 (21-88)	2003-2011	BMI < 24.9 (normal weight)/ 25<BMI<29.9 (overweight)/ BMI<BMI30 (obese)	64/77/42 (premenopausal) 45/68/60 (postmenopausal)	60/52/22 (premenopausal) 39/70/31 (postmenopausal)	7
O.Keskin	2013	Turkey	48.9±10.7 44.5±11.1/ 49.6±11.1/ 52.7±10.0	2001-2011	20-24.9 (normal weight)/ 25-29.9 (overweight)/ BMI≥30 (obese)	231/266/226	198/205/169	7
Geoffrey A. Porter	2006	Canada	60±15.5	2002.2.15- 2004.2.15	BMI <25 (normal/underweight)/ 25-29.9 (overweight)/ BMI≥30 (obese/severely obese)	36/33/46	130/144/130	8
Marianne Ewertz	2011	Denmark	---	1977-2006	BMI <25/ 25-29/ 30+	6867/3201/1489	4621/1937/849	7
Vincent C. Herlevic	2015	US	61.3 60.5/61.7/61.3	1997-2013	BMI<25 (normal weight)/ 25-30 (overweight)/ BMI>30 (obese)	40/71/142	47/79/144	8
Marian L. Neuhouser	2016	US	50-79	1993-1998	BMI<25 (normal weight)/ 25-30 (overweight)/ 30-35 (obese, Grade 1)/ BMI≥35 (obese, Grade 2+3)	168/245/184/138 (postmenopausal)	579/825/547/345 (postmenopausal)	8
G. Berclaz	2004	International Breast Cancer Study Group	48 (21-84)/ 53 (25-80)/ 55 (26-80)	1978-1993	BMI<24.9 (normal weight)/ 25.0-29.9 (intermediate)/ BMI≥30.0 (obese)	2613/1652/833	695/386/191	6
Vito Michele Garrisi	2012	Italy	---	2004-2006	BMI<24.9 (normal)/ 25-29.99 (overweight)/ BMI≥30 (obese)	43/63/38	63/38/24	6
Luca Mazzarella	2013	European Institute of Oncology	---	1995-2005	BMI <25 (under/normal weight)/ 25-29.99 (overweight)/ BMI≥30 (obese)	258/77/28 (ER positive) 149/66/29 (ER negative)	283/67/31 (ER positive) 159/63/18 (ER negative)	7
Amelia Smith	2018	US	67 (63,73)	1993-2009	BMI < 18.5 (underweight)/ 18.5-24.9 (normal weight)/ 25-29.9 (overweight)/ BMI≥30 (obese)	3/282/261/197 (postmenopausal)	19/869/819/561 (postmenopausal)	6
Kang Wang	2019	China	50.0±11.2 48.5±13.7/ 49.1±11.1/ 52.6±10.7	2005.1-2015.12	BMI<18.5 (underweight)/ 18.5-24.9 (normal weight)/ BMI≥25 (overweight and obese)	114/1644/537 (premenopausal) 70/1120/627 (postmenopausal)	100/1316/422 (premenopausal) 107/1184/559 (postmenopausal)	6
E.R. Copson	2014	UK	36 (18-40) 36 (18-40)/ 37 (18-40)/ 37 (24-40)	2000-2008	BMI<25 (under/healthy weight)/ 25-30 (overweight)/ BMI≥30 (obese)	736/419/284 (premenopausal)	766/354/236 (premenopausal)	7
Aruna Kamineni	2013	US	64.5 (40-93)	1988.1.1- 1993.12.31	BMI<25 (normal weight)/ 25-30 (overweight)/ BMI≥30 (obese)	32/27/12	174/102/66	6
Ronny Mowad	2013	US	49.8 53.2/49.1/49.3	1998.3-2011.9	BMI<25 (normal/underweight)/ 25-29.9 (overweight)/ BMI>30 (obese)	9/18/47	15/24/70	8
Foluso O. Ademuyiwa	2011	US	54 (26-92) 52.9/56.3/56.1	1996.7-2010.7	BMI≤24.9 (normal/underweight)/ 25-29.9 (overweight)/ BMI>30 (obese)	44/49/68	80/81/96	7
Shaheenah Dawood	2008	US	46 (23-76)/ 48 (23-78)/ 52 (28-78)	1974-2000	BMI≤24.9 (normal/underweight)/ 25-29.9 (overweight)/ BMI≥30 (obese)	186/175/186	21/19/16	7
Ozan Yazici	2015	Turkey	48 (18-92)	2002.1-2013.10	18.5-24.9 (normal weight)/ 25-29.9 (overweight)/ BMI≥30.0 (obese)	20/14/7 (premenopausal) 7/5/10 (postmenopausal)	549/393/226 (premenopausal) 228/419/409 (postmenopausal)	7

*BMI* Body mass index, *NOS* Newcastle-Ottawa's Scale

### Highest versus lowest BMI meta-analysis

**Fig. 2:**
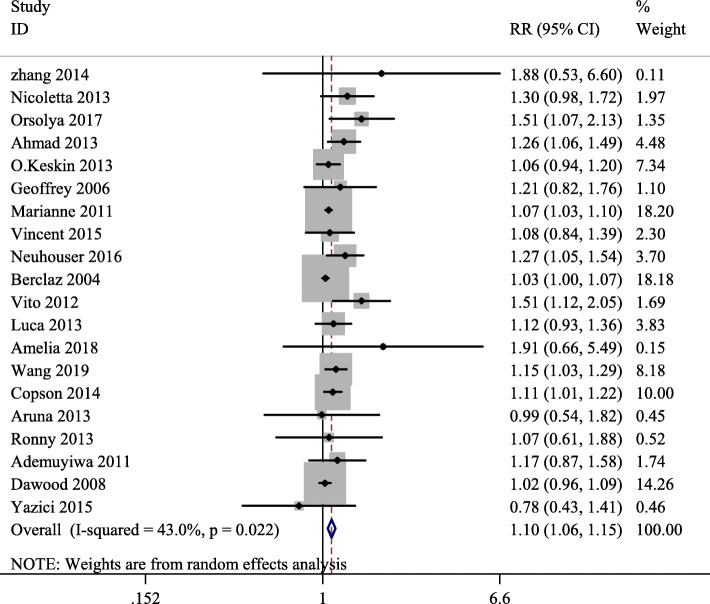
Forest plot of body mass index (BMI) and relative risk of lymph node metastasis for breast cancer (The highest versus lowest BMI categories are being compared, the summary relative risk was 1.10 (1.06–1.15), which showed a positive association between BMI and the risk of lymph node metastasis for breast cancer)

In this study, we selected the *RR*s corresponding to the highest BMI categories as the highest dose, and the *RR*s corresponding to the lowest BMI categories as the lowest dose. Heterogeneity among these 20 included articles was statistically significant (*P =* 0.022, *I*^*2*^ = 43.0%), and the random effect model was used for meta-analysis. The results showed that there was a link between BMI and the lymph node metastasis risk of breast cancer, with a summary *RR* of 1.10 (95%*CI*: 1.06–1.15) (Fig. 2).

### Subgroup analyses

When subgroup analyses were done for different areas, the results showed significant associations between BMI and lymph node metastasis of breast cancer in Asian (*RR* = 1.18, 95%*CI*: 1.08–1.30), European (*RR* = 1.08, 95%*CI*: 1.05–1.12) and American (*RR* = 1.13, 95%*CI*: 1.04–1.23) women. Interestingly, there were positive associations both in the premenopausal women (*RR* = 1.12, 95%*CI*: 1.04–1.20) and postmenopausal women (*RR* = 1.28, 95%*CI*: 1.14–1.44). Besides, we conducted a subgroup analysis stratified by study period, the *RR* (1.31, 95%*CI*, 1.14–1.50) of less than and equal to 5 years was prominent higher than that of more than 5 years (*RR* = 1.07, 95%*CI*: 1.05–1.10). For study population, positive significant associations between BMI and lymph node metastasis were observed in non-TNBC (*RR* = 1.08, 95%*CI*: 1.06–1.11), while poor association in TNBC patients (*RR* = 1.15, 95%*CI*: 0.88–1.49). The subgroup analyses are shown in Table 2.

**Table 2:** Subgroup analyses showing difference between studies included in the meta-analysis (highest versus lowest BMI)

Variables	Number of studies	Number of cases	Pooled RR (95%CI)	Test of heterogeneity		Publication bias	
				*I*^*2*^(%)	*P* value	Begg's *P* value	Egger's *P* value
All	20	30938	1.10 (1.06, 1.15)	43.0	0.022	0.538	0.003
Area							
Asia	3	2968	1.18 (1.08, 1.30)	0.0	0.555	1.000	0.339
Europe	8	19791	1.08 (1.05, 1.12)	44.6	0.082	0.266	0.116
America	8	3847	1.13 (1.04, 1.23)	47.4	0.065	0.902	0.079
Menopausal							
Pre	5	4291	1.12 (1.04, 1.20)	31.6	0.211	0.806	0.489
Post	6	4479	1.28 (1.14, 1.44)	0.0	0.865	0.452	0.656
Study period							
≤ 5y	6	2250	1.31 (1.14, 1.50)	0.0	0.709	0.707	0.860
> 5y	14	28688	1.07 (1.05, 1.10)	26.8	0.167	0.743	0.051
Study population							
TNBC	2	429	1.15 (0.88, 1.49)	0.0	0.789	1.000	---
Non-TNBC	18	30539	1.08 (1.06, 1.11)	48.2	0.012	0.363	0.003

*TNBC* Triple-negative breast cancer

### Dose-response analyses

**Fig. 3:**
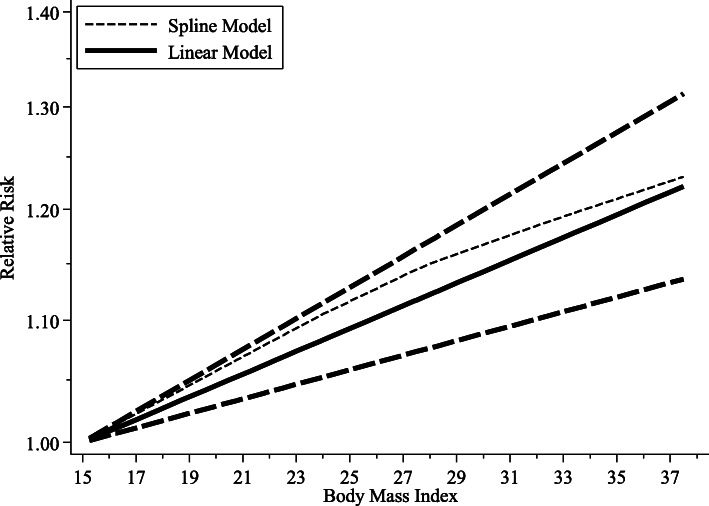
The linear association between body mass index (BMI) and lymph node metastasis for breast cancer (The solid line and the dash line represent the estimated relative risk (*RR*) and its 95% confidence interval (*CI*) for the fitted linear trend. Lines with short dashes represent the non-linear trend analysis result)

Figure 3 showed the results of linear and nonlinear dose-response analysis of BMI and relative risk of lymph node metastasis in breast cancer. Firstly, we conducted a regression model test (*P* = 0.465**)**, which showed no nonlinear dose-response relationship between BMI and lymph node metastasis. Secondly, linear dose-response regression model was used to test the relationship. The goodness of fit test (*χ*^*2*^ = 30.34, *P* = 0.048) showed there was heterogeneity among the studies, and the random-effect model was used for the meta-analysis. Regression model test (*χ*^*2*^ = 29.30, *P* < 0.001) revealed a positive linear dose-response association between BMI and lymph node metastasis. The results (*RR* = 1.01, 95%*CI*: 1.00–1.01) showed that for every 1 kg/m^2^ increment of BMI, the risk of lymph node metastasis increased by 0.89%.

The detailed information of the dose-response meta-analysis and subgroup analyses are shown in Table 3. In subgroup analyses, the results showed that the linear dose-response relationship between BMI and lymph node metastasis in Asian (*RR* = 1.01, 95%*CI*: 1.00–1.02), European (*RR* = 1.01, 95%*CI*: 1.00–1.01), American (*RR* = 1.01, 95%*CI*: 1.00–1.01), premenopausal (*RR* = 1.01, 95%*CI*: 1.00–1.03), postmenopausal (*RR* = 1.01, 95%*CI*: 1.01–1.02), study period ≤5 years (*RR* = 1.02, 95%*CI*: 1.01–1.03), study period > 5 years (*RR* = 1.01, 95%*CI*: 1.00–1.01) patients were statistically significant, and the risk increased by 0.99, 0.85, 0.61, 1.44, 1.45, 2.22, and 0.61%, respectively. And the results of other two subgroups (TNBC and non-TNBC) were missing because of too small sample size in TNBC.

**Table 3:** The results of linear dose-response analysis between body mass index (BMI) and lymph node metastasis of breast cancer

Variables	Number of cases	Test of heterogeneity	Model	Regression model test	*RR* (95%CI)
All	52904	χ 2=30.34, *P*=0.048	RE	χ 2=29.30, *P*<0.001	1.0089 (1.0057, 1.0122)
Area					
Asia	8854	χ 2=4.71, *P*=0.095	FE	χ 2=11.13, *P*=0.001	1.0099 (1.0041, 1.0157)
Europe	28979	χ 2=13.70, *P*=0.057	FE	χ 2=36.31, *P*<0.001	1.0085 (1.0057, 1.0113)
America	8701	χ 2=5.16, *P*=0.641	FE	χ 2=6.01, *P*=0.014	1.0061 (1.0012, 1.0110)
Menopausal					
Pre	8994	χ 2=10.53, *P*=0.032	RE	χ 2=5.61, *P*=0.018	1.0144 (1.0025, 1.0264)
Post	12456	χ 2=2.57, *P*=0.766	FE	χ 2=23.48, *P*<0.001	1.0145 (1.0086, 1.0204)
Study period					
≤ 5y	4901	χ 2=3.66, *P*=0.600	FE	χ 2=19.94, *P*<0.001	1.0222 (1.0124, 1.0321)
> 5y	48003	χ 2=16.61, *P*=0.218	FE	χ 2=40.88, *P*<0.001	1.0061 (1.0042, 1.0080)

*RE* Random effect, *FE* Fixed effect

### Sensitivity analysis

For the sensitivity analysis, we omitted one study at a time in turn to assess the potential studies which may influence the main results. The pooled *RR*s indicated little variation ranging from 1.09 (95%*CI*, 1.05–1.13) to 1.13 (95%*CI*, 1.06–1.19), and the result was not influenced by any single study, indicating that the meta-analysis result was stable.

### Publication bias

No publication bias was found for subgroup analyses, except for the overall studies using Egger’s test (*P* = 0.003) and studies on non-TNBC patients using Egger’s test (*P* = 0.003).

## Discussions

Dose-response meta-analysis results showed that there was a linear dose-response relationship between BMI and lymph node metastasis in breast cancer. For every 1 kg/m^2^ increment of BMI, the risk of lymph node metastasis increased by 0.89%. After grouping by areas, no significant geographical variation was detected, and the risk of lymph node metastasis increased by 0.99, 0.85, and 0.61% for every 1 kg/m^2^ increment of BMI in Asian, European, and American women, respectively. Higher proportions of overweight and obese black or African-American breast cancer patients in the United States were mentioned in Ronny’s study [45] and some other researches [49], which also tended to have poorer outcomes than white patients. An observation study of 223,895 women diagnosed with invasive breast cancer classified all patients into 8 race/ethnic groups including non-Hispanic white, Hispanic white, black, Chinese, Japanese, south Asian, other Asian, and other ethnicity [50]. Black women were significantly more likely to present with lymph node metastases than non-Hispanic white women (24.1% vs 18.4, *P* < 0.001), and lower probability was observed in Japanese women (14.6% vs 18.4%, *P* < 0.001). Whether this race/ethnicity disparity existed when BMI were assessed remained unknown, although confounding factors, such as socioeconomic status and treatment imbalance, contributed in part. Also, in Chinese Han women, a possible interaction between Interleukin-18-137G/C, −607G/T polymorphisms and BMI in breast cancer patients was identified [51]. Overweight and obese (BMI ≥ 24 kg/m^2^) patients with G/T genotype had a 5.45-fold (95%*CI*, 1.74–17.06) increased risk of lymph node metastasis relative to those with T/T homozygotes. Subgroup analyses grouped by race/ethnicity or genotype would be more accurate to explore the linkage between obesity and lymph node metastasis in breast cancer, unfortunately, which was not available in the selected studies.

Besides, the lymph node metastasis risk of breast cancer with BMI in premenopausal women (1.44%/1 kg/m^2^) was similar to that in postmenopausal women (1.45%/1 kg/m^2^). In postmenopausal patients, obese women would have a high concentration of circulating estrogen, since most estrogen is produced in the adipose tissue [52]. Moreover, in the peripheral adipose tissue, obese women have a high activity of aromatase enzyme, which converts androstenedione to estrogen and testosterone to estradiol in turn stimulated by both interleukin-6 (IL-6) and tumor necrosis factor-α (TNF-α) [53]. Elevated levels of estradiol are important to the development and growth of breast cancer, including lymph node metastasis, which are consistent with our results that shown increasing lymph node metastasis risk with BMI in postmenopausal women. Conversely, among premenopausal patients, systemic levels of estrogens are mainly produced by the ovaries, so not influenced by peripheral aromatization. It seems that obesity is not a independent factor in carcinogenesis and tumor metastasis in young breast cancer patients. Nevertheless, BMI was associated with a increased incidence for triple-negative subtype, but no association was shown in postmenopausal patients [54]. Similar findings also indicated that the association between obesity and TNBC was significant only among premenopausal women [55]. In addition to TNBC patients tended to present higher disease grade, more aggressive course, and high rate of recurrences [56], which may partly explained our results of similar lymph node metastasis risk in premenopausal and postmenopausal women. Due to small sample size in TNBC, subgroup analysis were not be conducted, as well as the interaction between triple-negative subtype and menopausal status. On the other hand, estrogen receptor (ER) positive in obese women also associated with menopausal status, although remained a matter of controversy in different studies [57, 58]. Only one included study [40] demonstrated results with ER positive and ER negative separately, and subgroup analysis was also failed.

When subgroup analysis was done for study period, it should be noted that a prominent increased risk (2.22%/1 kg/m^2^) of lymph node metastasis with BMI occurred in less than 5 years compared with more than 5 years (0.61%/1 kg/m^2^). A possible explanation is the apparent older participants (Table 1) in three included studies [34, 37, 44] followed less than 5 years, which constitutes approximately 80% of the subgroup patients. Another explanation is the substantial proportions (57–75%) of overweight and obese patients distributed in this subgroup, especially in large sample size study (75%) [37], which mainly resulted in higher lymph node metastasis risk in breast cancer patients.

Generally, lymph nodes involvement has been shown to predict for increased local and distant recurrence, as well as higher breast cancer mortality [59]. On basis of the Surveillance, Epidemiology, and End Results registry data, Brent’s [60] study found a significant association between large lymph node metastasis size and lower breast cancer-specific survival and overall survival even after controlling for other known prognosis factors including number of involved lymph nodes. Moreover, overweight and obesity are not only linked to breast cancer incidence, but women that are obese also have worse outcomes in terms of recurrence and survival. A clinical trial conducted in German [61] showed that obesity constituted an independent, adverse factor in patients with node-positive primary breast cancer. Women who were obese at the time of diagnosis had a shorter disease-free survival and overall survival as compared to women who were non-obese. Thus, BMI, as a modified risk factor, not only plays a crucial role in the occurrence of breast cancer, but also has adverse impact on the outcome and survival of patients. Similarly, we found that BMI had a great influence on the metastasis of various malignant tumors. For example, Zhihong Gong’s case-control study [62], following 752 middle-aged prostate cancer patients, concluded that obesity at the time of diagnosis was associated with an increased risk of developing prostate cancer metastasis, regardless of stage or primary treatment. Changhua Wu’s retrospective cohort study [63], enrolling 796 primary papillary thyroid cancer patients, indicated that the increment of BMI in patients was associated with the lymph node metastases, and other clinic-pathological features, such as tumor size, extrathyroidal invasion and so on.

It could be considered that the harm of tumor metastasis to patients should not be underestimated, but the reason was still unclear. Several hypothetical mechanisms could explain the association between obesity and lymph node metastasis in breast cancer. One is that the breast size of obese patients is larger, the adipose tissue is thicker, and the palpation of the primary tumor or enlarged axillary lymph nodes is more difficult. Therefore, the accuracy and sensitivity of ultrasonography, molybdenum target and other examinations will be reduced, leading to the delayed or even missed diagnosis of patients, so tumors often in advanced stage or have metastasized at the time of diagnosis [64]. Estrogen, most produced in adipose tissue, have a high level in obese or overweight women, via the aromatization of androstenedione to estrone and then converts to estradiol. This process would in turn facilitate tumor growth. In addition, leptin levels are also higher in obese individuals than those of normal weight, which related to tumor cell proliferation [65]. Some other adipocytokines, such as IL-6 and TNF-α released by activated macrophage, results in inflammation, which could be partly responsible for breast cancer development [66]. Other potential mechanisms for obesity-associated pathologic differences include higher insulin levels and insulin-like growth factors among obese women, which may increase estrogen levels and lead to higher proliferative rates [67]. Notably, in obese breast cancer patients, if the actual body surface area exceeds 2 m^2^, dose reductions during adjuvant chemotherapy are frequently applied [68]. Up to 40% of patients may receive limited chemotherapy doses that are not based on actual body weight to avoid possible side effects and toxicity [69]. Meanwhile, aromatase inhibitors, representing an effective endocrine treatment for hormone receptor positive breast cancer patients, were suspected to be less effective in suppression of estrogen levels enough to prevent recurrence in obese women regardless of menopausal status [70, 71]. Finally, obesity patients often have some unhealthy lifestyle habits, such as excess saturated fat intake and lack of physical activity, resulting in the accumulation of body acid cholesterol, trans fatty acid and other harmful lipid, which are recognized as risk factors for adverse prognosis of breast cancer.

Several limitations existed in our study. Firstly, BMI was calculated by measuring height and weight at the time of diagnosis, which was objective and avoided information bias to some extent. But long-term weight and body composition changes were not take into account, as well as some other potential modifiers (eg. waist circumference and waist-to-hip ratio) for the relationship of BMI and lymph node metastasis in breast cancer. Secondly, some included articles didn’t group BMI according to WHO standards, so the accuracy of the results would be affected in the highest versus lowest BMI meta-analysis. Thirdly, we didn’t have access to other key individual-level information except area, menopausal status, and study period, such as race, breast cancer sub-types, ER status, progesterone receptor (PR) status, human epidermal growth factor receptor 2 (HER2) status, and obesity associated risk factors (eg. dietary habits and physical inactivity), to examine the roles of these factors in lymph node metastasis. Finally, the retrospective nature of this meta-analysis could not be ignored, so the results should be interpreted with cautions.

## Conclusions

In conclusion, BMI significantly increases the lymph node metastasis risk of breast cancer. Overweight and obese breast cancer patients might benefit from adhering to a healthy lifestyle aiming at losing or controlling weight, as part of the comprehensive oncologic therapy. Further original studies are warranted to identify the link of BMI and lymph node metastasis in breast cancer.

## Data Availability

The datasets generated and/or analyzed during the current study are available in the manuscript.

## Abbreviations

BMI: Body Mass Index
OR: Odds ratio
PMC: PubMed Central
CNKI: The China National Knowledge Infrastructure
VIP: Chinese Scientific Journals
WanFang: Wanfang Data Knowledge Service Platform
NOS: Newcastle-Ottawa’s Scale
RR: Relative risk
TNBC: Triple-negative breast cancer
IL-6: Interleukin-6
TNF-α: Tumor necrosis factor-α
ER: Estrogen receptor
